# Exploring the correlation and mechanism of natural killer cell cytotoxic sensitivity against gastric cancer

**DOI:** 10.32604/or.2025.059426

**Published:** 2025-05-29

**Authors:** WENZHUO YANG, HAODONG CHEN, ZHILAN ZHANG, ZHIYONG XIA, YUANYUAN JIN, ZHAOYONG YANG

**Affiliations:** 1 The Department of Oncology, Beijing Hospital, Beijing, 100034, China; 2 NHC Key Laboratory of Biotechnology of Antibiotics, Institute of Medicinal Biotechnology, Chinese Academy of Medical Sciences, Beijing, 100050, China; 3 The School of Pharmacy, North China University of Science and Technology, Tangshan, 063210, China

**Keywords:** Umbilical cord blood natural killer (UCB-NK) cells, Oxaliplatin, Gastric cancer, Natural killer group 2 member D (NKG2D) ligand (NKG2DL), CD56 (neural cell adhesion molecule NCAM)

## Abstract

**Background:**

Human natural killer (NK) cells have attracted widespread attention as a potential adoptive cell therapy (ACT). However, the therapeutic effects of NK cell infusion in patients with solid tumors are limited. There is an urgent need to explore a suitable new treatment plan to overcome weaknesses and support the superior therapeutic activity of NK cells.

**Methods:**

In this study, the mechanisms underlying the susceptibility of gastric cancer (GC) cell lines AGS, HGC-27, and NCI-N87 to NK cell-mediated cytotoxicity were explored.

**Results:**

Lactic dehydrogenase (LDH) release assays showed that all three GC cell lines were susceptible to the umbilical cord blood NK (UCB-NK) cells, and HGC-27 cells with high CD56 expression were the most sensitive to UCB-NK, followed by NCI-N87 and AGS. When the expression of CD56 in HGC-27 cells decreased, the lytic activity of NK cells in HGC-27 cells was abating. In addition, combining oxaliplatin with NK cells produced additive anti-tumor effects *in vitro*, which may have resulted from oxaliplatin-induced NK group 2 member D (NKG2DL) upregulation in GC cells. These results of cytotoxicity activity showed that inhibition of CD56 expression might suppress the sensitivity of GC cells to NK cell-mediated cytotoxicity, and upregulation of the expression of NKG2DL on the surface of GC cells by oxaliplatin could enhance the killing sensitivity of NK cells.

**Conclusion:**

Collectively, our study provides a deeper theoretical foundation and a better therapeutic strategy for NK cell immunotherapy in the treatment of human GC.

## Introduction

Globally, gastric cancer (GC) was the fourth leading cause of cancer-related deaths in 2020, accounting for approximately 768,793 deaths [1,2]. Projections suggest a 62% increase in cases by 2040, totaling 1.77 million. The 5-year relative survival rates for different stages of gastric cancer vary significantly: for localized gastric cancer, the survival rate is approximately 75%; for regional gastric cancer, the rate drops to about 35%; and for metastatic gastric cancer, the survival rate is as low as 7% [3]. Chemotherapy can improve the survival rate of patients with advanced GC, and a combined regimen is better than monotherapy alone. In 2010, the European Union and the United States successively approved trastuzumab combined with chemotherapy as the first-line standard treatment of advanced or metastatic HER2-positive gastric/gastroesophageal junction adenocarcinoma. However, most GC patients lack sensitivity to trastuzumab in combination with chemotherapy.

As a breakthrough in cancer treatment, immunotherapy has become the most promising modality after surgery, chemotherapy, radiotherapy, and targeted therapy [4]. Cell-based anticancer immunotherapy using either T or NK effector cells has been used to treat malignancies, such as leukemia, melanoma, and renal cell carcinoma [5–8]. NK cells are crucial components of the innate immune system [9]and are capable of responding to different types of tumor cells and virus-infected cells without the need for prior antigen sensitization [10–12]. However, the effect of adoptive transfer NK cells alone on the treatment of solid tumors is not satisfactory. In clinical trials of colon, gastric, kidney, and lung cancer, there was no clinical response to the transfer of NK cells [13–15]. The low efficiency of clinical outcomes for solid tumors suggests that two parameters are critical for adoptively transferred NK cells to mediate therapeutically relevant effects: (1) intratumorally accumulation [16], and (2) persistence in an activated state. Therefore, effective strategies to circumvent these barriers may provide superior therapeutic activity.

There are various sources of NK cells used in clinical trials, including peripheral blood-derived NK cells, umbilical cord blood-isolated NK (UCB-NK) cells, umbilical cord blood CD34^+^ cell-derived NK cells, the NK-92 cell line, and iPSC-derived NK cells, each with its own advantages and disadvantages [17–21]. However, umbilical cord blood-derived NK cells recover well after cryopreservation and may be potential candidates for “off-the-shelf” therapy using primary NK cells [22]. In this study, we develop innovative strategies to generate high-purity UCB-NK cells in large numbers, without sorting or feeder cells. In addition, most amplified cells highly expressed functional markers, such as NK group 2 member D (NKG2D), DNAM-1, NKp30, NKp44, and CD16, and exerted strong cytotoxicity *in vitro* and in a xenograft model of human GC in NOG mice. Having successfully produced these high-quality NK cells, we demonstrated the mechanisms underlying the various sensitivities of different GC cell lines to NK cell cytotoxicity. Meanwhile, we revealed that oxaliplatin combined with NK cells induced additive anti-tumor effects *in vitro* by selectively up-regulating NKG2DLs in GC cells.

## Materials and Methods

All methods were performed in accordance with the relevant guidelines and regulations.

### Preparation of NK cells

UCB units were obtained at birth after normal full-term delivery with written informed consent. The use of UCB units was approved by the Medical Ethics Committee of ZIBO CHANGGUO Hospital (CGYJ-BD-080-V2). The UCB samples were stored at room temperature and processed within 12 h of collection. Pathogen-free evaluation of the separated cells was performed using a portion of the sample. To expand the rest of the sample, mononuclear cells (MNC) were isolated by Ficoll-Paque density gradient centrifugation. To generate UCB-NK cells, MNCs (between 1 × 10^6^ and 3 × 10^6^/mL) were cultured in a 175 cm^2^ flask with 50mL X-VIVO 15 (Lonza group Ltd., 02-060Q, Basel, Switzerland) supplemented with 5% auto-plasma, 1000 U/mL IL-2 (Beijing Four Rings Bio-pharmaceuticals Company, S19991010, Beijing, China) and 1 mL laboratory self-made activating factor including StemRegenin 1(2 µM) (Selleckchem, S2858, Houston, TX, USA), IL-15 (25 ng/mL) (PeproTech Inc., 200-15-100 ug, Waltham, MA, USA), and zoledronic acid (2 µg/mL) (Sigma-Aldrich, PHR1893, Missouri, Germany) for 4–5 days in a 37°C, 5% CO_2_ incubator. The cultured cells were centrifuged, and the supernatant was discarded. The cells were again cultured in X-VIVO 15 medium (Lonza, Basel, Switzerland) supplemented with 1000 U/mL interleukin (IL)-2 for 10–15 days. The cell density was checked and adjusted using a suitable amount of fresh medium every 2–3 days. At the end of the usual 14-day culture process, the purity of CD56^+^CD3^-^UCB-NK cells was >90%. For mycoplasma contamination tests of UCB-NK post-expansion, one-step mycoplasma PCR detection kits (Sigma-Aldrich, MP0050, Missouri, Germany) were used.

### Cell lines and culture

Human GC cell lines AGS, HGC-27, and NCI-N87 were purchased from the Basic Medical Institute, Chinese Academy of Medical Sciences (Beijing, China). All the gastric cell lines were cultured in Roswell Park Memorial Institute (RPMI) medium (Thermo Fisher Scientific, 11875093, Waltham, MA, USA), and supplemented with 10% fetal bovine serum (Thermo Fisher Scientific, A5670701, Waltham, MA, USA), 100 U/mL penicillin, and 100 μg/mL streptomycin (Thermo Fisher Scientific, 15140122, Waltham, MA, USA). The cells were grown as adherent cultures at 37°C in 5% CO_2_ and passaged after detachment using 0.05% trypsin. The mycoplasma detection method for all tumor cell lines was the same as that for UCB-NK cells. The 293T cell line was obtained from the Basic Medical Institute, Chinese Academy of Medical Sciences (Beijing, China) and was cultured in basic DMEM (Gibco, 11965092, Carlsbad, CA, USA) supplemented with 10% fetal bovine serum and incubated under a 5% CO_2_ atmosphere at 37°C.

### Flow cytometry

The cells were stained with antibodies for flow cytometry. Briefly, the cells were incubated with antibodies for 20 min at 4°C, then washed twice with PBS and analyzed using isotype antibodies as controls and a FACS Canto II (BD Biosciences, San Jose, CA, USA). Data were analyzed using ACEA Novo Express software (NovoExpress1.1.0, ACEA Biosciences Inc., San Diego, CA, USA). The following antibodies were used to stain single cell suspensions with the dilution ratio of 5 µL per million cells in 100 µL staining volume: FITC anti-human CD3 (UCHT1), APC anti-human CD56 (HCD56), PE anti-human CD314 (NKG2D) (1D11), APC anti-human CD340 (erbB2/HER-2) (24D2), APC Mouse IgG1, κ isotype Ctrl (MOPC-21), PE Mouse IgG1, κ isotype Ctrl (MOPC-21), PE anti-human MICA/MICB (6D4), PE anti-human CD336 (NKp44) (P44-8), PE anti-human CD337 (NKp30) (P30-15), and PE anti-human CD16 (3G8) (BioLegend, Inc., San Diego, CA, USA), and Human ULBP-1 PE-conjugated Antibody (170818), Human ULBP-2/5/6 PE-conjugated Antibody (165903), and Human ULBP-3 PE-conjugated Antibody (166510) (R&D System, Minneapolis, MN, USA).

### Plasmid construction and stable transfection

The YSH-LV001 vector (Ubigene Biosciences, Guangzhou City, China) was used to construct a specific CD56 shRNA-expressing plasmid. Two target sequences of the shRNA constructs that matched the requested genes were selected. The sequences were as follows: scramble- shRNA, CCTAAGGTTAAGTCGCCCTCG; shRNA1, AGAGGATGGAAACTCTATTAA; and shRNA2, CCGTTCCCTGAAACCGTTAAA. The accuracy of the recombinant vectors was confirmed by restriction enzyme digestion and sequencing analysis.

The 293T cells were obtained in the logarithmic growth phase and in good condition. After digestion and counting, the cells were seeded in a 10 cm dish at a concentration of 5 × 10^6^ cells/dish and then incubated until confluence reached 70% at 37°C and 5% CO_2_. Before transfection, the used culture medium was removed and the fresh culture solution was added. The lentivirus packaging and target plasmid (pCMV-VSVG: psPAX2: hNCAM1 shRNA-expressing plasmid = 5 μg: 15 μg: 10 μg) were added to a clean centrifuge tube, and a certain amount of Ubigene transfection reagent was added. The mixture was added dropwise to 293 T cells, and the dish was agitated to achieve uniform mixing. Approximately 6 h after the transfection, the culture medium containing the transfection system was removed and a fresh complete medium was added. After continuous cultivation for 72 h, the cell supernatant was collected, reconcentrated, and subpacked. The sample was then stored at −80°C for use in the preliminary determination of the biological virus titer by qPCR. HGC-27 cells were seeded at a density of 2 × 10^5^ cells/well in six-well plates and cultured for 24 h until they reached 50% confluence. Thereafter, the cells were treated with an appropriate amount of lentivirus. At 24 h post-transduction, the complete medium was replaced and transduction was continued for 48 h. Stable clones were selected with 4 μg/mL puromycin and maintained with 2 μg/mL puromycin.

### Quantitative real-time polymerase chain reaction (qPCR)

Total RNAs were isolated from cells dissolved in RNase-free water using an RNA simple Total RNA Kit (TianGen, DP419, Beijing, China); the A260/A280 ratio of the purified RNA was between 1.6 and 1.8. After reverse transcription, the synthesized cDNA samples were subjected to qPCR analysis. SYBR Green (Solarbio Science & Technology Co., Ltd., SY1020, Beijing, China) was used for qPCR, and specific primers were designed to ensure that the monitored genes had the same amplification efficiency and similar amplification product lengths. Data were analyzed by 2^−∆∆Ct^ method. The forward and reverse primers for amplifying hNCAM1 are as follows: 5′-CCAGTGCACCTAAGCTCGAA-3′ and 5′-CTCAGCATTCCAGTCCAGGG-3′. The amplified fragment was 203 bp in length. The β-actin with a 121-bp fragment was used as the internal control and was amplified with the following forward and reverse primers: 5′-CGAGCACGGCATCGTCAC-3′ and 5′-CTGGATAGCAACGTACATGGC-3′.

### Cell viability and cytotoxicity assay

According to the manufacturer’s protocol, the effect of oxaliplatin on tumor cell viability was assessed by Cell Counting Kit-8 (CCK8) (Dojindo, Kumamoto, Japan). In short, for the CCK8 assay, cells were seeded in 96-well plates (1 × 10^4^ cells/well) and then cultured at 37°C in 5% CO_2_. After 24 h of culture, the cells were treated with different concentrations of oxaliplatin and incubated for 24, 48, and 72 h. At the end of the incubation periods, 10 μL CCK8 solution was added to each well. The plates were then incubated for an additional 30 min. Finally, the absorbance was measured at 450 nm wavelength with a microplate reader (BioTek, Epoch 2, El Segundo, CA, USA).

The cytotoxicity of UCB-NK or UCB-NK combined with oxaliplatin was evaluated using the lactic dehydrogenase (LDH) release assay (Dojindo, Kumamoto, Japan). Target cells AGS, HGC-27(or CD56 gene knockout HGC-27 cell lines), and NCI-N87 were cocultured respectively with effector cells at different effector to target (E/T) ratios in 96-well plates with a final volume of 200 μL at 37°C and 5% CO_2_. After 4 h, the supernatant was collected on a new plate. Absorbance was measured at 450 and 600 nm using a microplate reader (BioTek, USA). The percentage of specific lysis at each E/T ratio was calculated.

### Xenograft model using HGC-27 cell line

HGC-27 cells (1 × 10^6^) were injected subcutaneously into the right scapula of 4–5-week-old female NOG mice (n = 20, Beijing Vital River Laboratory Animal Technology, Beijing, China). When the tumor volume reached 50–100 mm^3^, the HGC-27-bearing mice were intraperitoneally injected with oxaliplatin (5 mg/kg) every other day or UCB-NK cells (5 × 10^6^ per mouse) twice a week. The first administration of oxaliplatin was recorded on day 0, and UCB-NK cells were injected two days after oxaliplatin administration. The control group was administered PBS. The mice were raised under conditions without specific pathogens, and their health was closely monitored. After the tumor volume reached 2000 mm^3^, the mice were euthanized, and tumors were separated and measured (tumor volume (mm^3^) = (major axis) * (minor axis)^2^/2). The study was approved by the Institutional Animal Care and Use Committee of Beijing Vital River Laboratory Animal Technology Co. (No. P2021101) and was performed in accordance with the Animal Management Rules of the Ministry of Health of the China and the ARRIVE guidelines.

### Statistical analysis

Each assay was set up in triplicate wells and repeated three times at least. Data were analyzed using GraphPadPrism 8.0 software (GraphPad Software Inc., San Diego, CA, USA). Student’s *t*-test was used to evaluate the significance of differences between the two groups, and a *p*-value < 0.05 was considered statistically significant.

## Results

### Identifying the total number and cell types of UCB-NK following ex vivo expansion

**Figure 1:**
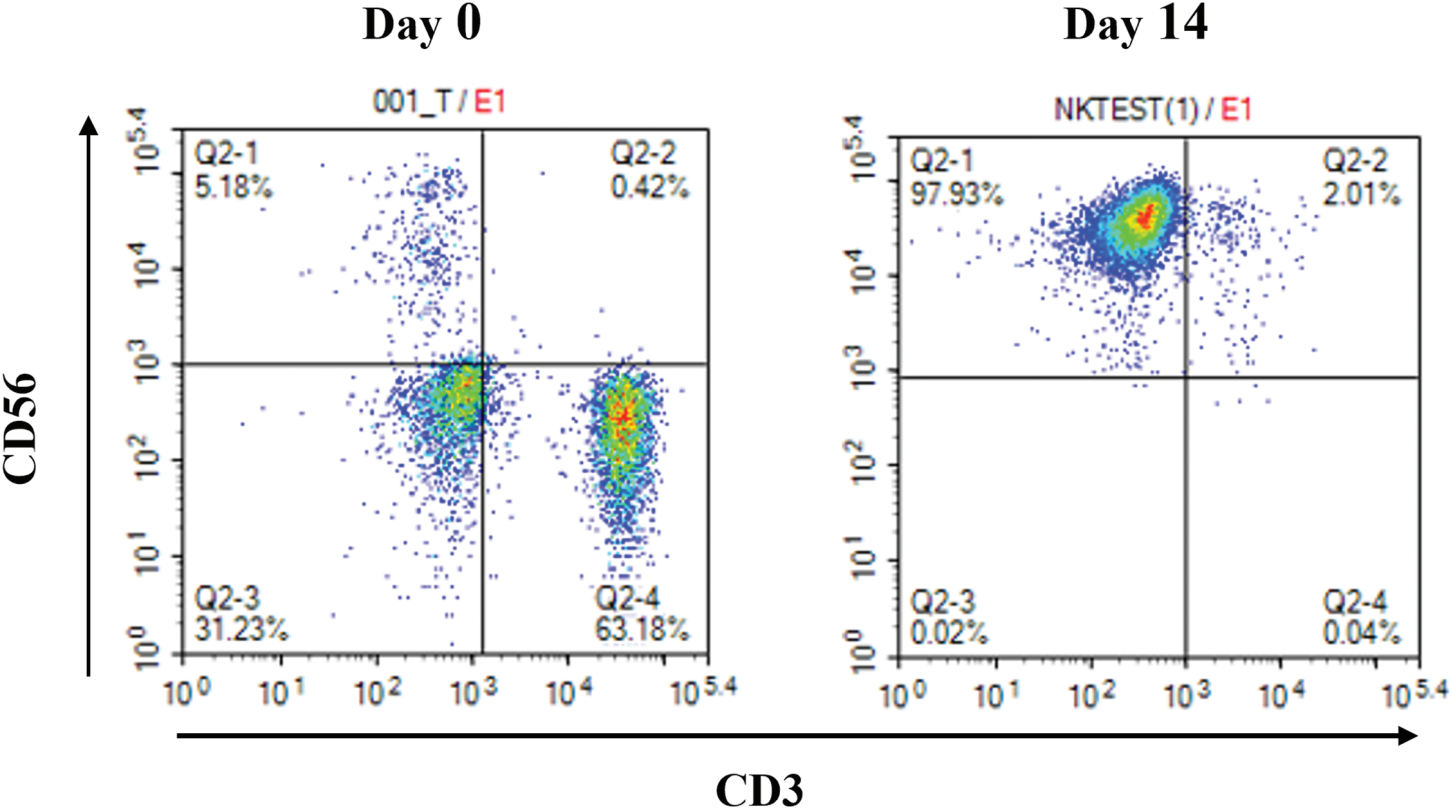
Representative flow cytometric scatter of CD56 and CD3 double staining. Percentage of CD3^-^CD56^+^-expressing NK cells in umbilical cord blood mononuclear cells cultured for 0 days and 14 days.

**Figure 2:**
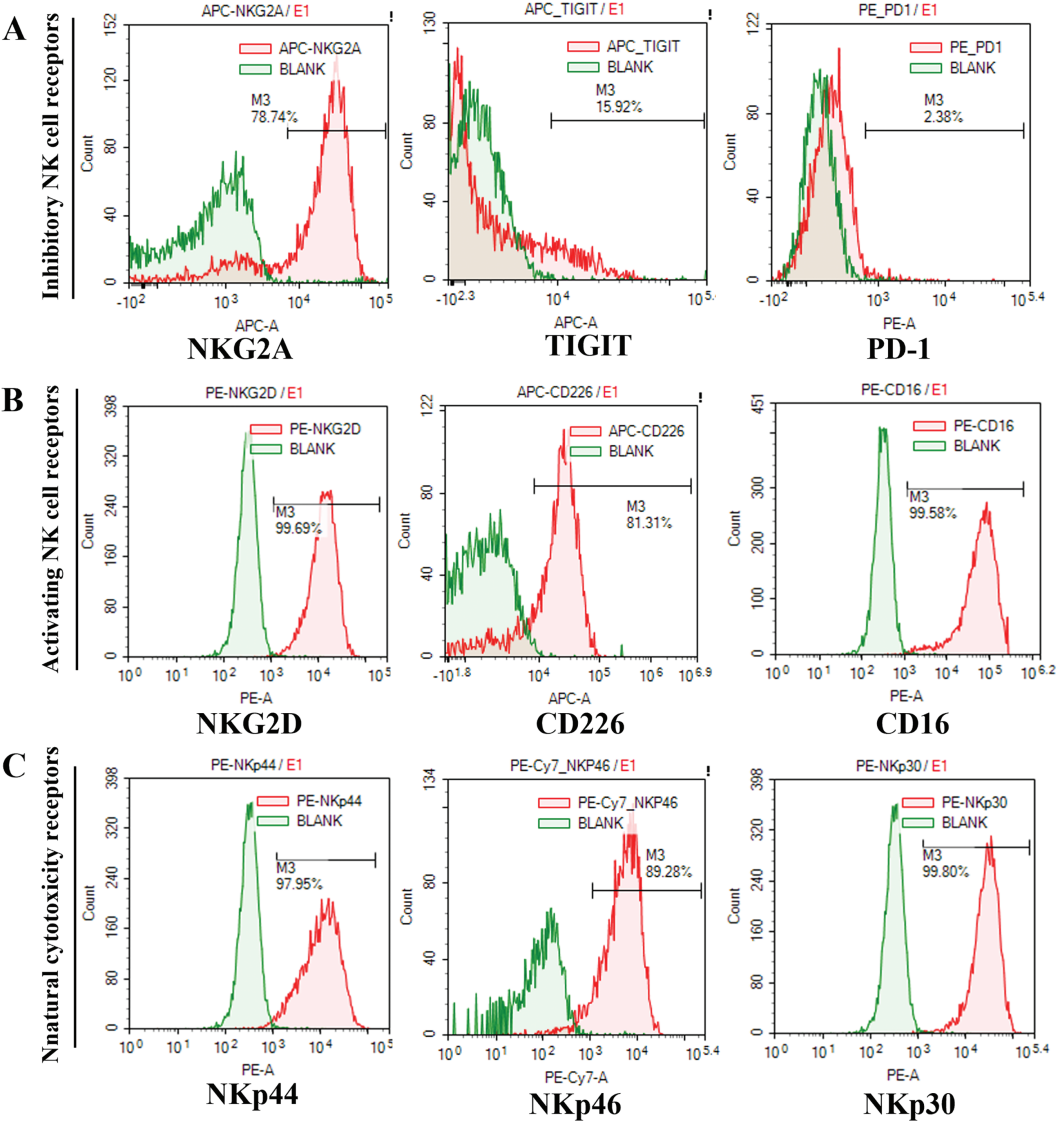
Flow cytometry analysis of the UCB-NK cell phenotype. (A) NKG2A, TIGIT, and PD-1 expression on CD3^-^CD56^+^ NK cells. (B) NKG2D, CD226, and CD16 expression on CD3^-^CD56^+^ NK cells. (C) NKp44, NKp46, and NKp30 expression on CD3^-^CD56^+^ NK cells.

After 14 days of culture, the total number of NK cells by expansion using this method increased approximately 5500-fold compared to that of initial NK cells in PBMCs isolated from every 80–100 mL UCB units (5.12 × 10^6^ ± 0.09 × 10^6^ *vs*. 5.9 × 10^9^ ± 0.07 × 10^9^). The proportion of CD3^-^CD56^+^ cells in the initially isolated MNCs and cultured UCB-NK was determined using flow cytometry. The ratio of CD3^-^CD56^+^cells in the initially isolated MNCs was 5.13 ± 0.33%, but it increased in cultured UCB-NK to 96.77 ± 0.86%, indicating that NK cells were preferentially expanded compared to other types of cells under the given culture condition (Fig. 1). The expression of activating, natural cytotoxicity, inhibitory, and checkpoint receptors on UCB-NK cells was examined using flow cytometry. As shown in Fig. 2A–C, the expression of inhibitory receptor NKG2A and checkpoint receptors TIGIT and PD-1 was 83.47 ± 3.65%, 15.71 ± 1.43 and 2.50 ± 0.14, respectively. The expression of activating receptor NKG2D, CD226, and CD16 in the cultured UCB-NK was 96.71 ± 4.02%, 82.26 ± 3.55 and 98.37 ± 1.22, respectively. Finally, The expression of natural cytotoxicity receptors, NKp30, NKp44, and NKp46, was 99.04 ± 1.00%, 97.90 ± 0.45%, and 88.65 ± 1.88%, respectively.

### Examining the expression of surface molecules related to NK sensitivity in gastric cancer cell lines

**Figure 3:**
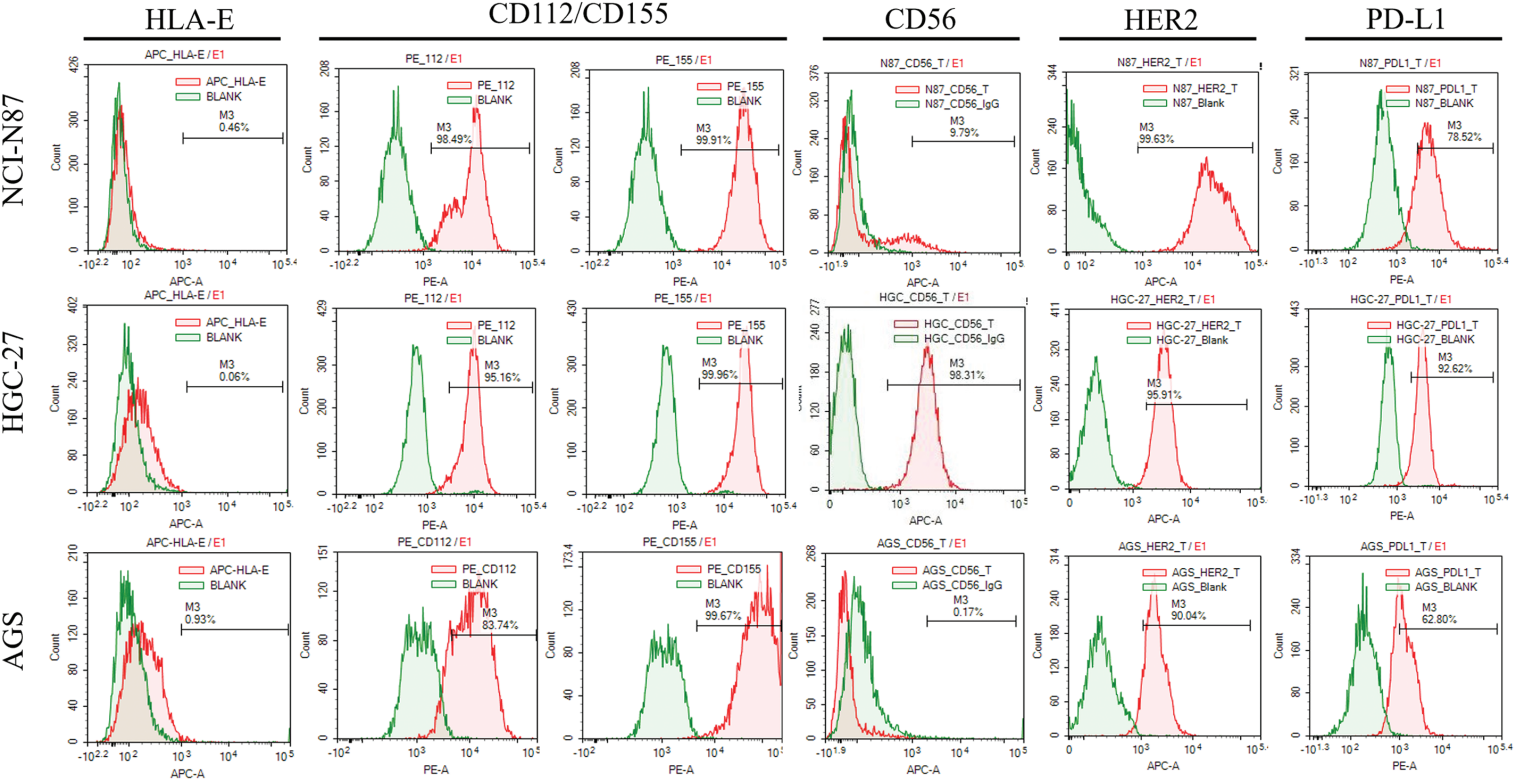
The expression of surface molecules (CD56 and HER2) and NK cell receptors-ligands (HLA-E: ligand of NKG2A receptor; CD155: ligands of DNAM-1/TIGIT/CD96 receptor; CD112: ligands of DNAM-1/TIGIT) in gastric cancer cell lines was determined using flow cytometry.

To test the relationship between the expression of surface molecules and the sensitivity of GC cells to UCB-NK cell cytotoxicity, we first used flow cytometry to quantify the protein expression of HER2, CD56, and PD-L1 in three GC cell lines. There was an evident variation in expression; for instance, NCI-N87 cells with high expression of HER2 and PD-L1 showed only weak CD56 expression, but HGC-27 cells exhibited high expression of the surface molecules HER2, CD56, and PD-L1. AGS cells showed high expression of both HER2 and PD-L1 and the absence of CD56 expression (Fig. 3). We then evaluated the expression of DNAM-1(CD226) ligands CD155 and CD112 and NKG2A ligands HLA-E. In general, CD112 and CD155 were clearly expressed in all three cell lines, and all three cell lines showed no HLA-E expression (Fig. 3).

We evaluated the effect of oxaliplatin on activating ligands of NK cell receptors (NKG2D) in GC cells using flow cytometry. Primarily, HGC-27 cells lacked ULBP expression and showed a certain amount of MICA/B expression, whereas NCI-N87 cells exhibited a certain amount of ULBP2 and MICA/B expression and lacked ULBP1 and ULBP3 expression. AGS cells lacked MICA/B and ULBPs expression. After treatment with low concentrations of oxaliplatin for 48 h, HGC-27 cells displayed higher expression of MICA/B (Fig. S1), while there was no significant impact on ULBPs. NCI-N87 cells treated with oxaliplatin showed an increase in ULBP2 expression but no obvious changes in MICA/B, ULBP1, or ULBP3 expression (Fig. S2). In AGS cells, there was no up-regulation of MICA/B or ULBPs (Fig. S3).

### Oxaliplatin enhances NK cell-killing sensitivity to GC cells

**Figure 4:**
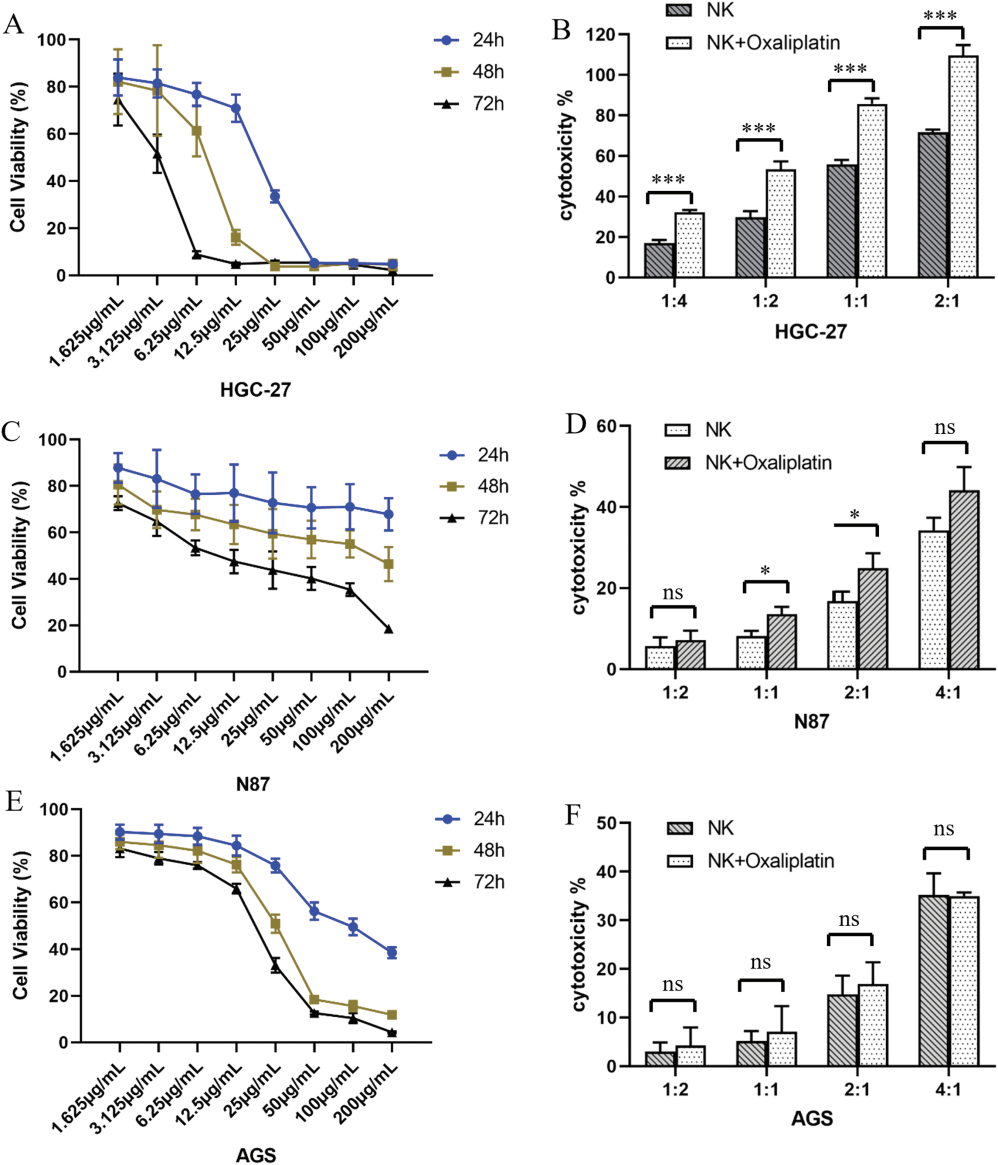
Oxaliplatin inhibited the proliferation of gastric cancer cells. Human gastric cancer cell lines HGC-27 (A), NCI-N87 (C), and AGS (E) were treated with different concentrations of oxaliplatin over a period of 24, 48, and 72h, and total viable cell counts were determined by CCK-8 assay. Cytotoxicity of UCB-NK cells against gastric cancer cell lines and oxaliplatin pretreated gastric cancer cell lines HGC-27 (B), NCI-N87 (D), and AGS (F) was measured by the lactic dehydrogenase (LDH) release assay at different effector to target ratios (E/T). Statistical analysis was done by two-tailed Student’s *t*-test, error bars denote S.D. *p < 0.05; ***p < 0.001; ns, no significance.

The effect of oxaliplatin on GC cell viability was determined using the CCK8-based cell viability assay. With the increase in drug concentration and extension of action time, the viability of all three GC cell lines significantly decreased. Although oxaliplatin had certain effects on the viability of all three GC cells, it had the greatest impact on HGC-27 compared with the other two cell lines, AGS and NCI-N87 (Fig. 4A,C,E).

The cytotoxicity of UCB-NK against all three GC cells (AGS, HGC-27, and NCI-N87 cells) was determined by LDH-based cytotoxicity assay. Cytotoxicity against GC cells increased significantly with the increasing E:T ratio. Although all three GC cell lines responded to the cytotoxicity of UCB-NK, HGC-27 was the most sensitive to UCB-NK, followed by NCI-N87 and AGS (Fig. 4B,D,F).

We subsequently determined the effects of oxaliplatin on UCB-NK cell-mediated cytotoxicity, GC cells were pretreated with low-concentration oxaliplatin (3.125 μg/mL) for 48 h before co-culture with UCB-NK cells that were derived from the same donor. HGC-27 cells pretreated with oxaliplatin showed significant cytolysis of UCB-NK cells. A relatively weak trend was observed for NCI-N87 cells, with increased specific lysis at effector-to-target ratios (E/T) 1:1 and 2:1. However, this phenomenon was not observed in AGS cells, indicating that oxaliplatin was unable to increase the susceptibility of AGS cells to NK cell-mediated cytotoxicity (Fig. 4B,D,F).

### Stable knockout of CD56 in HGC-27 cells mediated by shRNA

**Figure 5:**
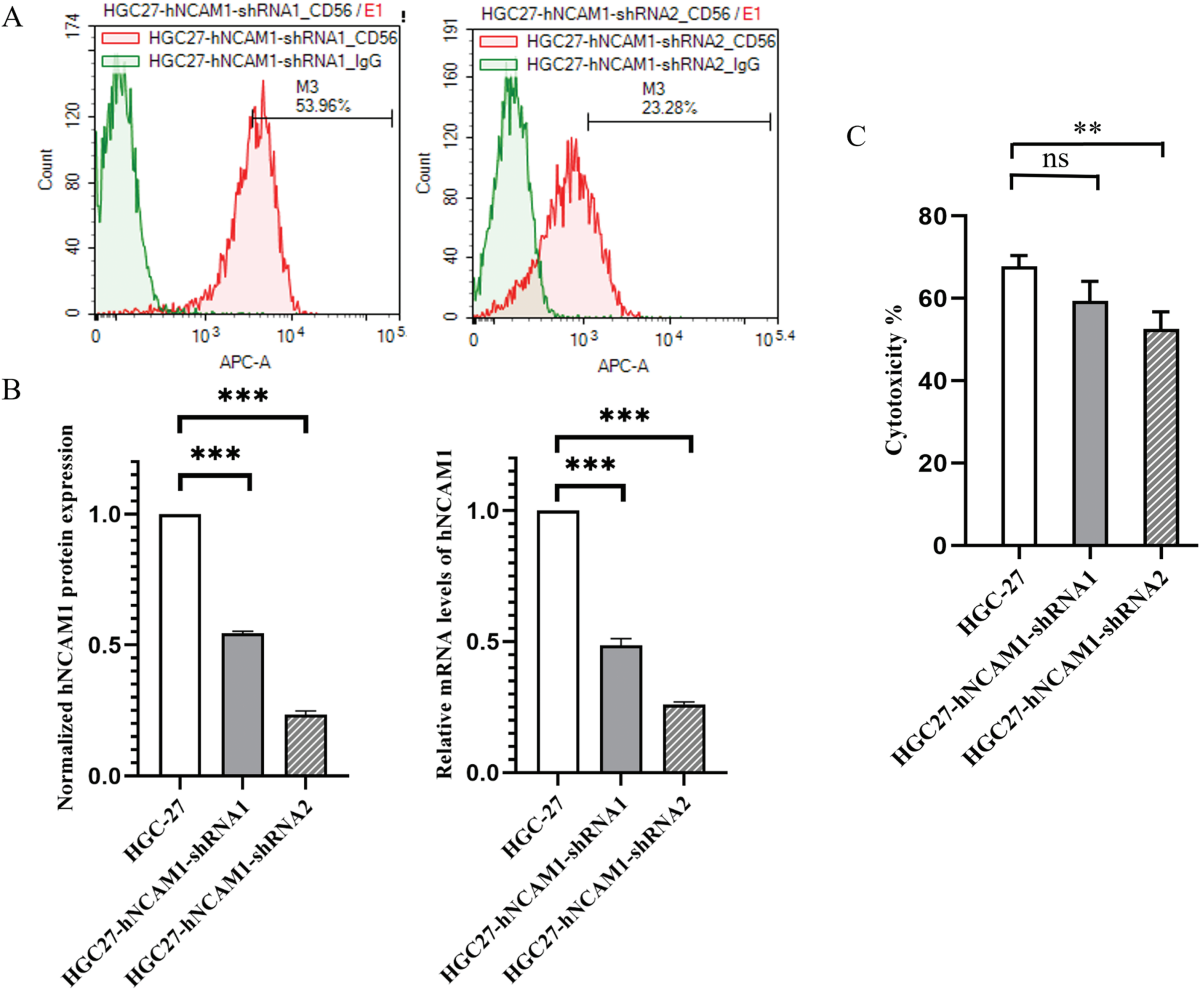
shRNA-mediated inhibition of CD56 expression in HGC-27 cells. (A) The protein expression levels of CD56 in HGC-27 cells were determined by flow cytometry. (B) Normalized protein expression of CD56 in HGC-27 cells and specific shRNA-treated cells; Relative mRNA levels of CD56 in HGC-27 cells and specific shRNA-treated cells determined by quantitative real-time PCR analysis. (C) Cytotoxicity of UCB-NK cells against gastric cancer cell lines HGC-27 and specific shRNA-treated HGC-27 cells was measured by the lactic dehydrogenase (LDH) release assay at an effector to target ratio (E/T) of 2:1. ** *p* < 0.01; *** *p* < 0.001; ns, no significance.

To investigate the role of CD56 in human GC cells, plasmid-mediated RNAi technology was used to inhibit the endogenous expression of CD56 in HGC-27 cells. The protein and mRNA expression levels of CD56 in normal and plasmid-transfected HGC-27 cells were determined using flow cytometry and real-time PCR, respectively. As shown in Fig. 5A, the protein expression of CD56 was 53.96% and 23.28% in CD56-specific shRNA1-and shRNA2-treated cells, respectively. The current data showed that CD56 protein expression was significantly reduced by 45.4% and 76.5% in CD56-specific shRNA1-and shRNA2-treated cells as compared with that in HGC-27 cells. (Fig. 5B). Similarly, compared with the relative mRNA level of the HGC27 cell line, that of the HGC27-CD56-shRNA1 cell line was downregulated by 54.0%, and that of the HGC27-CD56-shRNA2 cell line was downregulated by 73.1% (Fig. 5B). These results indicated that a specific shRNA targeting CD56 could effectively inhibit the expression of CD56 in HGC-27 cells at both the transcriptional and translational levels.

### The effect of CD56 knockdown on natural killer-mediated cytotoxicity to HGC-27 cells

Regarding the expression of surface proteins in GC cells, the most significant difference was observed in CD56 expression. We investigated whether the expression of CD56 is related to the sensitivity of different GC cells to NK cell-mediated cytotoxicity. HGC-27 cells with the highest expression of CD56 were used to construct tumor cell lines with different degrees of CD56 knockdown. Then, the cytotoxicity of UCB-NK against both HGC-27 cells with shRNA-mediated interference targeting-theCD56 gene and the original HGC-27 cells was determined by an LDH-based cytotoxicity assay at an effector to target ratio (E/T) of 2:1. As shown in Fig. 5C, when the expression of CD56 in the target cells decreased, the lytic activity of NK cells on the target cells was abrogated.

### Investigating UCB-NK-mediated killing following ex vivo expansion in a xenograft model

**Figure 6:**
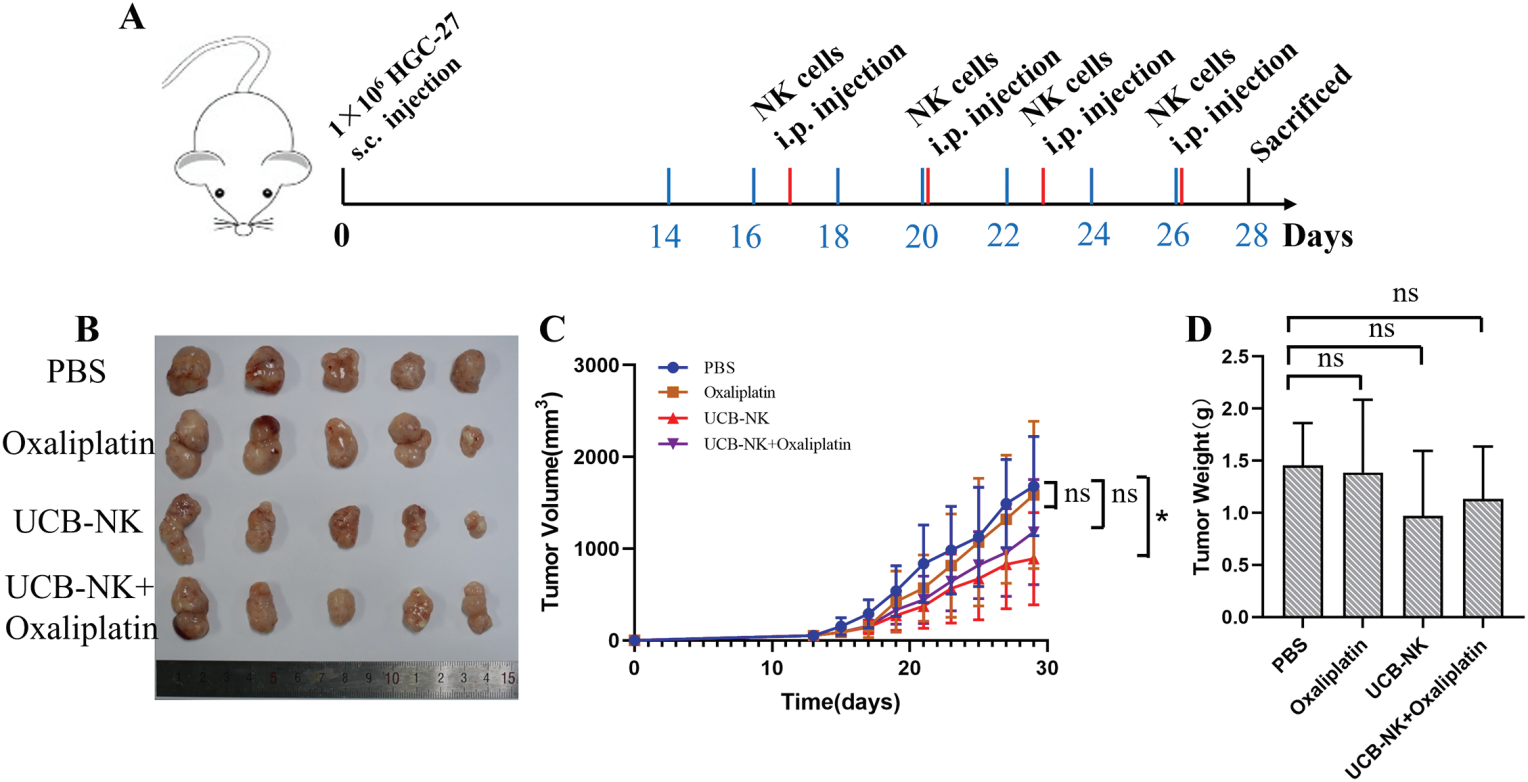
Effects of UCB-NK cells and oxaliplatin on xenotransplantation of human gastric cancer HGC-27 cells in NOG mice. (A) Schematic diagram of the treatment program of the HGC-27-bearing mice. (B) At the end of the experiment, resected tumors from each group were collected and imaged. (C) Tumor growth curves during the treatments. *p < 0.05; ns, no significance. (D) Tumor weights for each group were measured on the last day of the experiment; ns, no significance.

A xenograft model was established by the subcutaneous injection of HGC-27 cells into NOG mice to evaluate the antitumor ability of UCB-NK cells *in vivo*. Fourteen days after tumor cell injection, the tumor-bearing mice began to receive treatment. Tumor growth was monitored by regularly measuring the tumor dimensions using calipers (Fig. 6A). In NOG mice engrafted with human HGC-27 cells and treated with UCB-NK cells, the tumor volume was significantly reduced compared to that in mice treated with PBS. In contrast, there was no dramatic difference in tumor size between NOG mice that received PBS and those on low-dose oxaliplatin, or between NOG mice that received PBS and those that received low-dose oxaliplatin combined with NK cells. Moreover, compared to PBS, tumor weights were not remarkably reduced following UCB-NK cell treatment, low-dose oxaliplatin, or low-dose oxaliplatin combined with NK cells (Fig. 6B,C,D). Taken together, these results show that UCB-NK cells have enormous potential to eliminate cancer in a subcutaneous GC model.

## Discussion

Recently, immunotherapy has emerged as an alternative to conventional therapies for treating advanced refractory cancers. NK cells are innate lymphocytes, that are the first line of defense against infection and surveillance of tumors [23–25]. A previous study [26] evaluated the number of NK cells in 72 patients with gastric adenocarcinoma and its relationship with survival. Compared to patients with a low number of NK cells, patients with a high number of NK cells had a higher survival rate, especially in advanced GC. The data obtained by Saito et al. [27] indicated that the apoptotic frequency of NK cells in GC patients was remarkably higher than that in normal controls. These data indicate that the enhanced quantity and function of NK cells are closely related to a longer survival time [24]. However, a major obstacle in the development of NK cell-based therapies is the lack of effective methods for generating a sufficient number of activated NK cells for clinical applications. Owing to the challenges of *ex vivo* expansion and activation of NK cells, the development of an optimized method for preparing NK cells that can be applied clinically is crucial for achieving NK cell-based immunotherapies. To this end, we developed a relatively simple but effective method to expand and activate umbilical cord blood-derived NK cells (UCB-NK), which do not require feeder cells and can be differentiated and efficiently expanded up to 5500-fold, resulting in the production of a high-purity UCB-NK product with a high cytolytic capacity. Our data also indicated that UCB-NK has anti-cancer potential against GC both *in vitro* and *in vivo*.

*In vitro* studies have shown that UCB-NK exhibits cytotoxicity against all three GC cell lines, namely HGC-27, N87, and AGS cells, and increases with increasing E: T ratios. However, UCB-NK cell-mediated cytotoxicity varied among the three GC cell lines. To explore this, we tested the expression of various surface proteins in tumor cells. The results showed that the most significant difference was in the expression of CD56 and ligands of the NKG2D receptor (NKG2DLs). To our knowledge, more than 90% of human tumor cells express at least one type of NKG2DL on their surface, while they are almost unexpressed in healthy tissues, indicating that cancer progression must be related to the biogenesis of NKG2DLs in cancers [28]. In this study, we found that HGC-27, which is the most sensitive to NK cell killing, expresses the NKG2D ligand MICA/B, whereas AGS does not express any NKG2D ligands and has the least sensitivity. Indeed, there is a clear correlation between the expression levels of NKG2D-ligand on tumor cells and their sensitivity to NK cells. We also tested the effect of oxaliplatin on the tumor-killing activity of NK cells. We found that compared to the untreated group, NK cells in the oxaliplatin-pretreatment group had stronger killing effects on HGC-27 and N87 cells. Further analysis indicated that the expression of MICA/B on HGC-27 cells was enhanced by oxaliplatin pre-treatment, and the expression of ULBP-2 on N87 cells also increased. Based on these findings, we believe that oxaliplatin enhances the killing activity of NK cells against tumor cells by altering the expression of NKG2DLs on tumor cells.

CD56 is a surface protein belonging to the Ig superfamily of cell adhesion molecules (CAMs) and is also known as a neural CAM (NCAM). A few studies have suggested that CD56 may also have a functional role with inconsistent or even opposite conclusions [29,30]. Here, we examined the impact of CD56 expression on NK-cell-mediated killing and found no linear correlation between varying degrees of decreased CD56 (hNCAM1) expression and the sensitivity of HGC-27 cells to NK cells. However, when the expression of CD56 decreased significantly, the lytic activity of NK cells towards the target cells was also remarkably reduced. Therefore, different expression levels of CD56 did not seem to be significantly correlated with NK cell-mediated killing of the tumor target cells used in our analysis; however, very low or no expression of CD56 on tumor cells was likely to affect the cytotoxicity of NK cells.

An *in vivo* study using HGC-27 further validated the effectiveness of UCB-NK and UCB-NK combined with the oxaliplatin in the treatment of GC, UCB-NK treatment at a 2 day-interval notably inhibited tumor growth in volume, but there was no significant difference in tumor weight. Regrettably, a remarkable reduction in tumor volume and weight was not observed in the UCB-NK combined with oxaliplatin group as compared with mice treated with PBS. It can be seen that when the tumor grows to a certain volume or is in an advanced stage, the effectiveness of UCB-NK treatment is limited. This may be because NK cells cannot effectively infiltrate the tumor interior. Therefore, it may be necessary to further rationally redesign and/or combine other treatments, such as IL-2 or IL-15 to support the survival of NK cells in the body based on dose optimization in future studies, and cell therapy may be more suitable for postoperative adjuvant treatment in solid tumors. A limitation of the current study is that we did not use HGC-27 cells stably knocked out by CD56 to establish a xenograft model and conduct *in vivo* anti-tumor activity research.

## Conclusions

In conclusion, this study identified NKG2DLs as a predictive biomarker of GC sensitivity to NK-mediated cytotoxicity. Moreover, oxaliplatin selectively induced the upregulation of NKG2DL expression on GC cell HGC-27 and N87, thus activating the NKG2D/NKG2DL receptor-ligand pathway, and enhancing the tumor cell-killing effects of NK cells *in vitro*. Our study provides a theoretical basis and specific clinical applications for GC with the expression of NKG2DLs.

## Supplementary Materials

Supplementary Figure 1 The expression of ligands of NKG2D in HGC-27 gastric cancer cell lines was determined by using flow cytometry. Statistical analysis was done by two-tailed Student’s *t*-test, error bars denote S.D. *p < 0.05; ns, no significance

Supplementary Figure 2 The expression of ligands of NKG2D in N87 gastric cancer cell lines was determined by using flow cytometry. Statistical analysis was done by two-tailed Student’s *t*-test, error bars denote S.D. *p < 0.05; ns, no significance.

Supplementary Figure 3 The expression of ligands of NKG2D in AGS gastric cancer cell lines was determined by using flow cytometry. Statistical analysis was done by two-tailed Student’s t-test, error bars denote S.D. ns, no significance.

## Data Availability

All data generated or analyzed during this study are included in this published article and its supplementary information files.

## Supplementary Materials

The supplementary material is available online at https://doi.org/10.32604/or.2025.059426.
