# Supplementary figures and images for: Exploring the correlation and mechanism of natural killer cell cytotoxic sensitivity against gastric cancer
Source: Oncol Res. 2025 May 29;33(6):1485–94. doi: 10.32604/or.2025.059426 (PMC12144607; doi:10.32604/or.2025.059426)

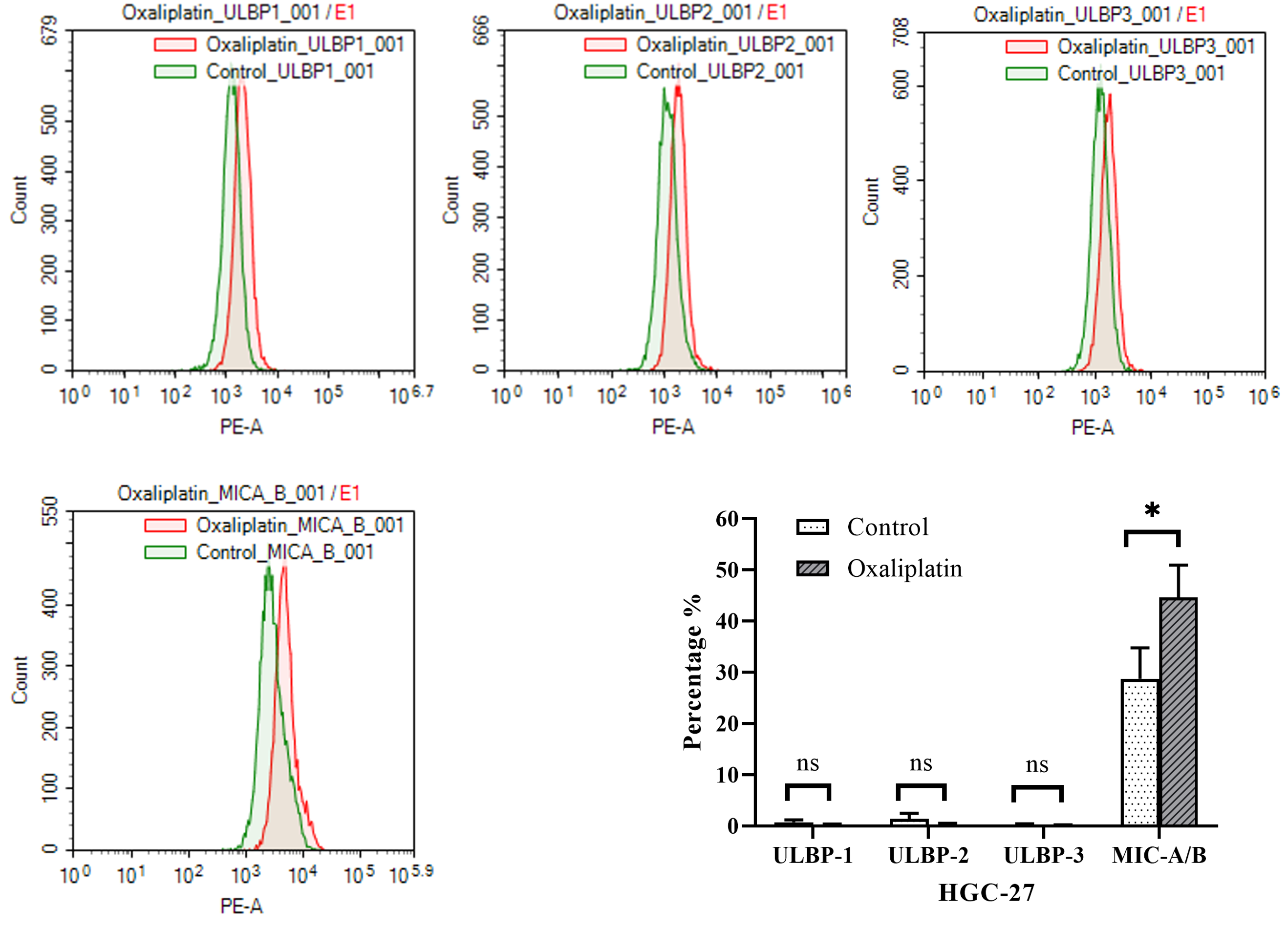

Supplement: Supplementary Figure 1 [file OncolRes-33-59426-s001.tif]

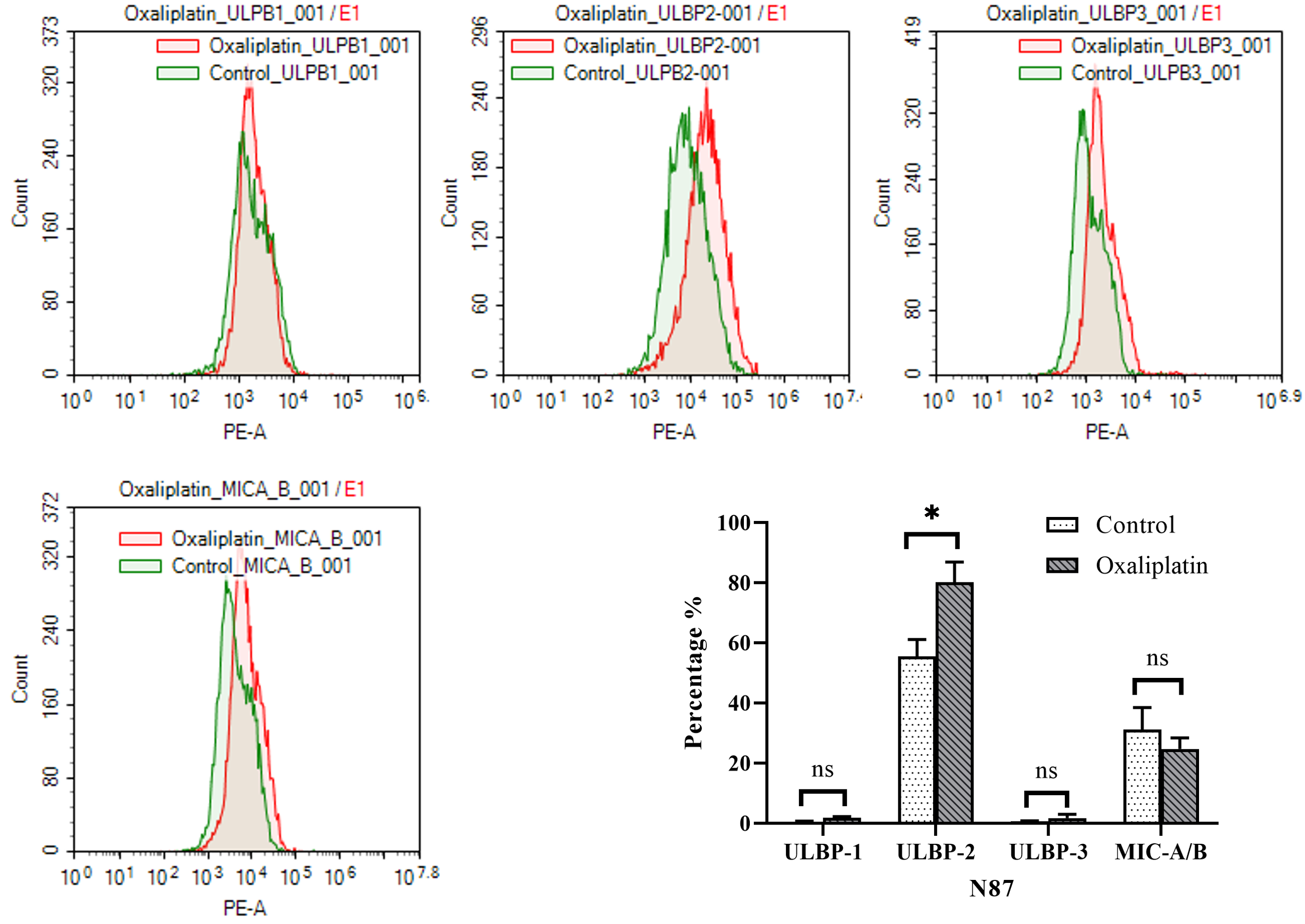

Supplement: Supplementary Figure 2 [file OncolRes-33-59426-s002.tif]

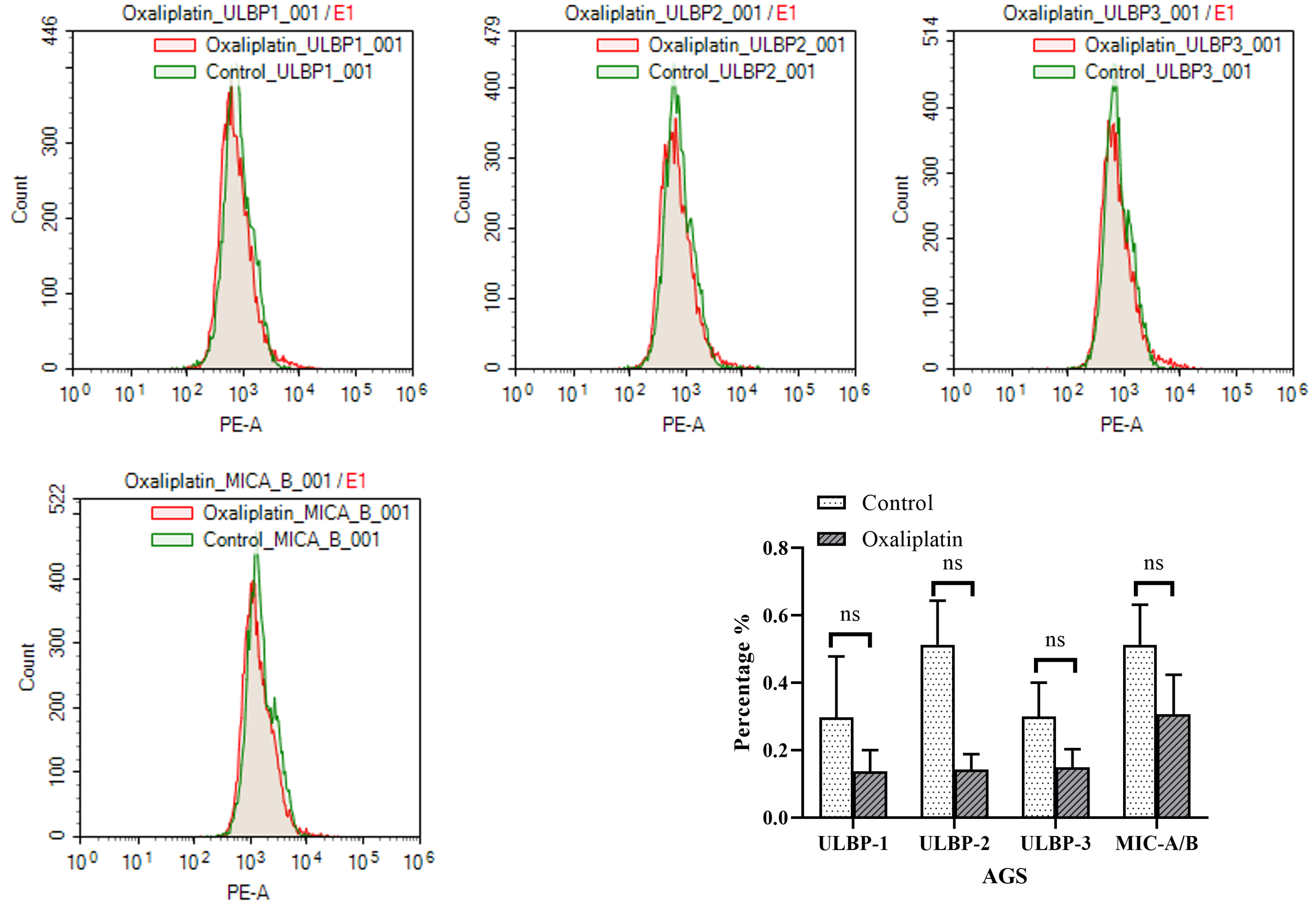

Supplement: Supplementary Figure 3 [file OncolRes-33-59426-s003.tif]
